# Oxidative stress in neurodegenerative disease: causation or association?

**DOI:** 10.18632/oncotarget.14650

**Published:** 2017-01-13

**Authors:** Jeremy M. Van Raamsdonk, Irving E. Vega, Patrik Brundin

**Affiliations:** Laboratory of Aging and Neurodegenerative Disease, Center for Neurodegenerative Science, Van Andel Research Institute, Grand Rapids, MI, USA

**Keywords:** oxidative stress, Huntington’s disease, Parkinson’s disease, Alzheimer’s disease, aging, Neuroscience

Reactive oxygen species (ROS) are highly reactive oxygen containing molecules that can cause oxidative damage to the basic building blocks of the cell including DNA, protein and lipids. To defend against ROS-mediated damage organisms express antioxidant enzymes such as superoxide dismutase (SOD). Oxidative stress occurs when the generation of ROS exceeds the capacity to detoxify ROS. Based on the observation that oxidative damage is increased in many neurodegenerative conditions, it has been proposed that elevated oxidative stress is an important step in the pathogenesis of these (as well as other) diseases. However, the overarching question remains as to whether oxidative stress is the cause or consequence of neurodegeneration.

Huntington’s disease (HD) is a polyglutamine toxicity disorder caused by a trinucleotide CAG repeat expansion in the HD gene. A role for oxidative stress in HD was proposed due to the increased oxidative damage detected in postmortem tissues and animals models of the disease. While multiple compounds with antioxidant properties have been shown to be beneficial in mouse models of HD (creatine, coenzyme Q10, α-lipoic acid, ethyl eicosapentanoic acid, Vitamin C), antioxidants have shown little or no efficacy in the treatment of patients with HD.

In our recent work, we interrogated the role of oxidative stress in HD using the powerful genetics of *C. elegans* [1]. As in other animal models of HD, we found that HD worm models exhibit increased oxidative stress and multiple phenotypic deficits resulting from polyglutamine toxicity. To determine the role of oxidative stress in the development of the polyglutamine toxicity phenotypes, we increased oxidative stress in HD worm models through the deletion of *sod* genes, or decreased oxidative stress through treatment with antioxidants. While these interventions modulated oxidative stress as predicted, they had no impact on the onset or severity of polyglutamine toxicity phenotypes. With the caveat that these experiments were completed in *C. elegans* models and not human HD, the results suggest that while oxidative stress is increased in HD, it does not contribute significantly to disease pathogenesis. This conclusion is consistent with the fact that no genes encoding antioxidant enzymes have been identified as genetic modifiers of HD, and the fact that treatment with antioxidants has failed to show benefit in HD patients.

Our previous work has examined the role of oxidative stress in aging. The Free Radical Theory of Aging proposes that ROS generated by normal metabolism are the primary cause of aging. To test this theory, we used a genetic approach and found that increasing oxidative stress through the deletion of *sod* genes does not result in decreased lifespan [2], even when all of the *sod* genes are deleted [3]. In fact, we found that under certain conditions increasing ROS levels can extend longevity [4]. This suggests that while oxidative stress increases with age, it does not cause aging.

Alzheimer’s disease (AD) is the most prevalent type of dementia, affecting more than 5 million individuals in the US. As with HD, oxidative stress has been proposed to contribute to the pathogenesis of AD [5, 6]. Biometals including iron, zinc and cooper, which are mediators of ROS formation, have been directly associated with conformational changes in Amyloid β (Aβ) peptides, leading to the accumulation of toxic fibrillary Aβ aggregates. Oxidative stress has also been shown to cause tau hyperphosphorylation and aggregation. Multiple genetic risk factors for AD such as APOE Ɛ4, CD2AP, TOMM40 and TSPO are thought to cause increased oxidative stress through mitochondrial dysfunction. Variants of genes that play protective roles against oxidative stress such as CLU, GSTO2, MTFR1 and MSRB3 were also identified as risk factors for AD. Despite the vast genetic and biochemical evidence demonstrating that oxidative stress is increased in AD, and the fact that treatment with antioxidants has been effective in animal models, the use of antioxidants as a therapeutic strategy has failed to show benefits in AD patients. Thus, it remains to be determined if oxidative stress plays a causative role or it is induced as part of the pathophysiology of AD.

Parkinson’s disease (PD) is the second most common neurodegenerative disorder after AD, and affects around 1 million Americans. Clinically, it is characterized by hypokinesia, rigidity, and tremor, as well as several non-motor symptoms. Neuropathologically, late-stage PD patients exhibit loss of the majority of dopamine neurons from the substantia nigra with a corresponding decrease in striatal dopamine, and intraneuronal alpha-synuclein aggregates (Lewy pathology) in widespread brain areas. Numerous findings have implicated increased ROS in the PD pathogenic process including increased oxidative damage to lipids and protein [7, 8]. The substantia nigra has high iron and dopamine concentrations, both of which could promote oxidative stress. Findings in sporadic PD, and from rare genetic forms (DJ-1, PINK1, parkin), have directly implicated mitochondrial dysfunction in PD, which can also cause increased ROS production. While several studies in experimental PD models suggest that antioxidant therapies can interfere with pathogenic mechanisms, these findings have yet to translate to therapies that slow neurodegeneration in the clinical disorder.

**Figure 1:**
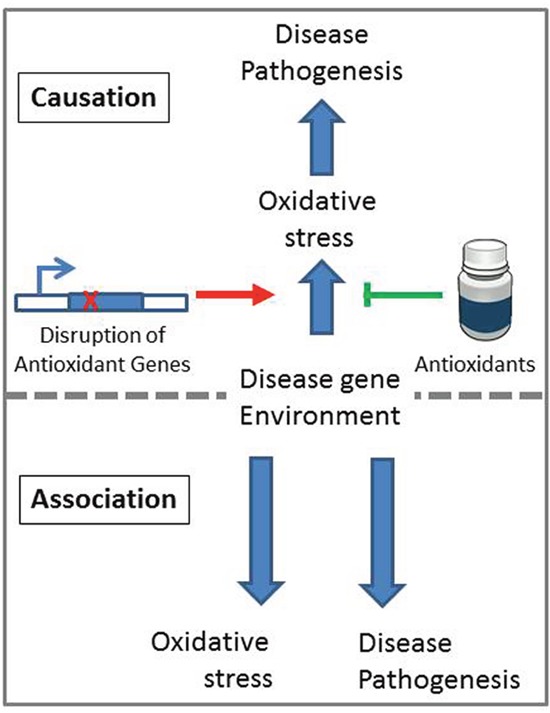
Causation and association models for the role of oxidative stress in neurodegenerative disease If oxidative stress contributes to disease pathogenesis (top) then increasing oxidative stress through activity-lowering single nucleotide polymorphisms in antioxidant genes, or decreasing oxidative stress through treatment with antioxidants should affect the onset and or severity of the disease. If oxidative stress is associated with disease (bottom) then the disease gene, or environmental factors causing the disease, give rise to oxidative stress and disease symptoms independently.

Overall, oxidative stress has been shown to be increased in multiple neurodegenerative diseases and aging. The fact that treatment with antioxidants has failed to show benefit in clinical trials and that core antioxidant enzymes such as SOD have not been identified in genome wide association studies suggests that the association between oxidative stress and disease may not be causative. Nonetheless, to definitively distinguish between association and causality, it is important to directly test the effect of modulating oxidative stress on disease pathogenesis (Figure 1).
